# Evaluation of relative effect potencies (REPs) for dioxin-like compounds to derive systemic or human-specific TEFs to improve human risk assessment

**DOI:** 10.1007/s00204-016-1724-9

**Published:** 2016-05-09

**Authors:** Karin I. van Ede, Majorie B. M. van Duursen, Martin van den Berg

**Affiliations:** Division of Toxicology and Veterinary Pharmacology, Institute for Risk Assessment Sciences (IRAS), Utrecht University, P.O. Box 80.177, NL-3508 TD Utrecht, The Netherlands

**Keywords:** Dibenzofurans, Dioxins, Human risk assessment, PCBs, TEF-concept

## Abstract

Toxic equivalency factors (TEFs) are generally applied for estimating human risk of dioxins and dioxin-like compounds using systemic (e.g., blood) levels, even though these TEFs are established based on intake doses in rodent studies. This review shows that systemic relative effect potencies (REPs) can deviate substantially from intake REPs, but are similar to in vitro-derived REPs. Interestingly, the in vitro REPs for 1,2,3,4,6,7,8-heptachlorodibenzo-*p*-dioxin (HpCDD) and 2,3,4,7,8-pentachlorodibenzofuran (4-PeCDF) are up to one order of magnitude higher than their in vivo REPs and WHO-TEFs, based on oral intake. In addition, clear species-differences in in vitro REPs were apparent for some congeners. Especially the human-derived REP for polychlorinated biphenyl 126 is one to two orders of magnitude lower than rodent REPs and its current WHO-TEF. Next, suggested adapted systemic or human-specific TEFs for these congeners were applied to calculate changes in systemic TEQ concentrations in studies from the USA, Germany and Japan and compared with either the JECFA TDI or USEPA RfD of TCDD. Overall, the effect of such TEF changes for these three congeners on total TEQ roughly balances each other out in the general population. However, results may be different for situations in which a specific group of congeners dominates. For those congeners that show a distinct deviation between either intake and systemic REPs or between rodent- and human-based in vitro REPs, we propose that especially REPs derived from human-based in vitro models are weighted more heavily in establishing systemic or human-specific TEF values to improve human health risk assessment.

## Introduction

Assessing the potential risk associated with exposure to dioxin-like compounds is challenging, as humans and wildlife are exposed to a complex mixture of these structurally related compounds (Safe 1994a). Based on the assumption that they share the same mechanism of action and data from experimental studies, it is accepted that their individual toxicities are additive. This has led to the development of the toxic equivalency concept (Safe 1990, 1994b), in which each congener is assigned a specific toxic equivalency factor (TEF) that reflects its potency to produce an aryl hydrocarbon receptor (AhR)-mediated biological or toxicological effect compared with the most potent congener, 2,3,7,8-tetrachlorodibenzo-*p*-dioxin (TCDD). To characterize the total toxicity in a matrix, such as food, total toxic equivalencies (TEQs) can be calculated by multiplying the concentration of each congener with its TEF value, after which it is summed up to calculate total TEQs. This approach is now generally used for risk characterization in food, feed and human populations.

From the early 1990s, the World Health Organization (WHO) started organizing international expert meetings with the objective of harmonizing TEFs for dioxin and dioxin-like compounds (DLCs). In 1993, the first evaluation was done that resulted in human and mammalian WHO-TEFs (Ahlborg et al. 1994). Since the second reevaluation in 1998, WHO-TEFs have been distinguished between mammals, birds and fish, with mammalian WHO-TEFs being used for human risk assessment (Van den Berg et al. 1998). In June 2005, a third WHO expert meeting was held to reevaluate the mammalian 1998 WHO-TEF values. For the latter meeting, a database with all known in vivo and in vitro relative effect potencies (REPs) was compiled that formed the basis of the present WHO-TEFs (Van den Berg et al. 2006; Haws et al. 2006). Because these TEFs are derived from a range of REPs using various endpoints and bioassays, the WHO expert panels from 1998 and 2006 estimated that these TEFs are surrounded by at least an order of magnitude uncertainty (Van den Berg et al. 1998, 2006). Currently, there are 7 polychlorinated dibenzo-*p*-dioxins (PCDDs), 10 polychlorinated dibenzofurans (PCDFs) and 12 dioxin-like polychlorinated biphenyls (PCBs) that have been assigned with a WHO-TEF value (see Tables 1 or 2).

Despite the large amount of supporting toxicological data on DLCs, some crucial gaps still exist in the present TEF methodology for these compounds. One major concern is whether the current WHO-TEFs, which are primarily based on in vivo studies with oral dosage, can also be used for human risk assessment when based on a systemic concentration, e.g., blood. Several studies already showed that for some congeners, blood-based REPs could be significantly different from those based on oral (intake) doses. This suggests that these “intake” REPs for hazard characterization might lead to misinterpretation of the risk when applied to blood concentrations (DeVito et al. 1997, 2000; van Ede et al. 2013, 2014a). Another important potential pitfall in the current WHO-TEFs comes from the fact that these are mostly based on rodent studies, but are ubiquitously applied for human risk assessment. Both uncertainties have been explicitly acknowledged during the most recent WHO-TEF expert meeting, and the expert panel specified the need to evaluate whether systemic as well as human-specific TEFs would result in a more accurate human risk assessment for DLCs (Van den Berg et al. 2006).

In this review, we address the question to what extent the use of current rodent-derived “intake” TEFs provides accurate results for human risk assessment if blood or tissue levels are used. To address this issue, we firstly assess whether in vitro-derived REPs can be used as a surrogate for systemic REPs. Secondly, we calculate the impact of adapted TEFs, based on our evaluation, on human risk estimates using existing data and reference values.

## In vitro-derived REPs as predictors for in vivo systemic REPs

**Fig. 1:**
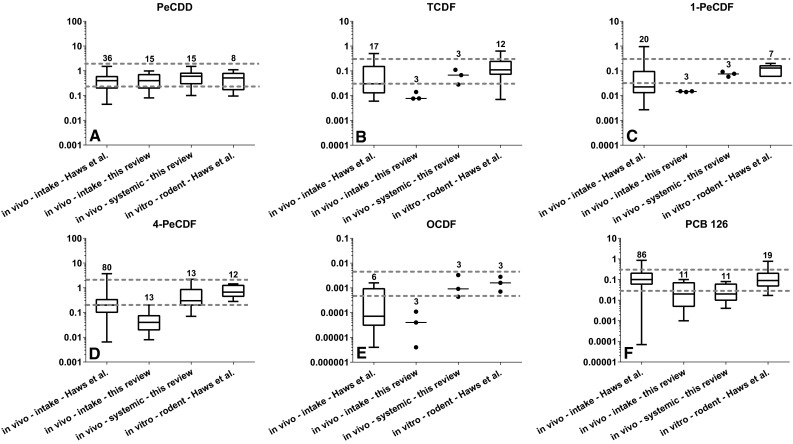
Boxplot comparison of in vivo REPs based on an administered dose (intake) or systemic concentration (blood plasma or skin) and in vitro-derived REPs for PeCDD (**a**), TCDF (**b**), 1-PeCDF (**c**), 4-PeCDF (**d**), OCDF (**e**) and PCB 126 (**f**). The boxplots; in vivo—intake—Haws et al. and in vitro—rodent—Haws et al. represent data from the 2004 REP database (Haws et al. 2006). The boxplots; in vivo—intake—this review and in vivo—systemic—this review represent data from one to four different studies in which the potency of the congener is determined based on either the administered (intake) dose or a systemic (plasma or skin) concentration (DeVito et al. 1997, 2000; Van Ede et al. 2013, 2014a). The *number* above the boxplot represents the number of REPs. The* gray dotted lines* represent the ± half log uncertainty area around the median in vitro REP

Theoretically, it may very well be possible that results from in vitro studies with DLCs are a better reflection of the actual potency of a congener determined at the target tissue in in vivo studies, as in both situations, the toxicokinetic properties of a congener, such as absorption, distribution, metabolism and elimination, are not playing a major role of concern. In other words, in vitro-derived REPs may better reflect in vivo REPs based on a systemic concentration. In this respect, studies using primary cultures and cell lines of rodent but in particular human cells may provide relevant information that can be used for human risk assessment if based on systemic concentrations. To assess whether indeed in vitro-derived REPs are more comparable to systemic REPs, we combined intake REPs and in vitro REPs from the 2004 REP database (Haws et al. 2006) with the very few in vivo studies in which intake REPs as well as systemic REPs were determined (Haws et al. 2006; DeVito et al. 2000; Van Ede et al. 2013, 2014a). These data are presented in Fig. 1.

In Fig. 1, the comparison between the different REPs is shown for six congeners: 1,2,3,7,8-pentachlorodibenzo-*p*-dioxin (PeCDD), 2,3,7,8-tetrachlorodibenzofuran (TCDF), 1,2,3,7,8-pentachlorodibenzofuran (1-PeCDF), 2,3,4,7,8-pentachlorodibenzofuran (4-PeCDF), 1,2,3,4,6,7,8,9-octachlorodibenzofuran (OCDF) and 3,3′,4,4′,5-pentachlorobiphenyl (PCB 126). For each congener, two dotted lines are shown in the graph representing a half log uncertainty area around the median in vitro REP. These values are based on the suggested minimal uncertainty around an established WHO-TEF (Van den Berg et al. 2006). However, it should be recognized that this ± half log uncertainty was suggested by the WHO panel based on expert judgment and not on actual statistical analysis for different congeners. Consequently, the ± half log uncertainty used in our review purely functions to illustrate deviations from the median in vitro REP and its uncertainty range from other types of REPs. Based on this comparison, it is apparent that for TCDF, 1-PeCDF, 4-PeCDF and OCDF the in vivo REPs are lower than the systemic REPs and in vitro REPs (see Fig. 1). It can be noted that for these four congeners the toxicokinetics are very different compared to the reference congener TCDD. The congeners TCDF and 1-PeCDF are much more rapidly metabolized and eliminated than TCDD. In addition, due to the large molecular size, OCDF is more poorly absorbed from the gastrointestinal (GI) tract than TCDD (Birnbaum and Couture 1988; Chen et al. 2001; Birnbaum et al. 1980; Van Den Berg et al. 1989; Brewster and Birnbaum 1988). Finally, 4-PeCDF is sequestered in the liver to a much higher degree than TCDD due to its high affinity binding to the CYP1A2 protein (Brewster and Birnbaum 1987; Chen et al. 2001; Devito et al. 1998). As a consequence, systemic REPs for these congeners based on a skin or plasma concentration are found to be higher when compared to the intake REPs (Fig. 1b–e) (DeVito et al. 1997; Van Ede et al. 2013, 2014a). For these congeners, it is clear that the systemic REPs are closer to the in vitro REPs than to the intake REPs. Such similarities are less distinct for PeCDD and PCB 126 (Fig. 1a, f, respectively). For PCB 126, the median systemic REP is somewhat lower than the median in vitro REP. However, it should be noted that also the intake REPs from these studies were lower than the WHO-TEF and those reported in the 2004 REP database (see Fig. 1f). Together, these results suggest that in vitro REPs can potentially be a good representative for systemic REPs, which may allow us to use the large amount of available in vitro data on DLCs to evaluate systemic *versus* intake differences in relative potencies.

## The role of toxicokinetics and species-differences in REPs

As described earlier, each step in toxicokinetics generally affects the in vivo REP of a congener, if it behaves significantly differently from TCDD. Moreover, species-specific differences in toxicokinetics and toxicodynamics of DLCs can also influence the REP of a congener. Some species-differences in AhR-mediated responses can clearly be attributed to genetic differences. Generally, the human AhR is considered to be relatively less responsive to DLCs than the rodent AhR (Ema et al. 1994; Connor and Aylward 2006). However, if the difference in potency between species is similar for TCDD and a specific congener, this obviously does not lead to species-differences in relative effect potency. Nonetheless, distinct species-specific differences in REPs have been described for some PCDDs and PCDFs, such as 4-PeCDF, 1,2,3,4,7,8-hexachlorodibenzofuran (4-HxCDF), 1,2,3,6,7,8-hexachlorodibenzofuran (6-HxCDF), 1,2,3,4,6,7,8-heptachlorodibenzo-*p*-dioxin (HpCDD) and 1,2,3,4,7,8,9-heptachlorodibenzofuran (9-HpCDF) (Sutter et al. 2010; Nagayama et al. 1985; Van Ede et al. 2014b; Larsson et al. 2015), but particularly the species-difference in toxicity of the non-*ortho*-substituted PCB 126 has been subject of much scientific debate (Nagayama et al. 1985; Silkworth et al. 2005; Sutter et al. 2010; Zeiger et al. 2001; Van Duursen et al. 2003, 2005; Van Ede et al. 2014b; Larsson et al. 2015).

In order to evaluate congener- and species-specific differences in various REPs between humans and rodents, we combined REP data that were published since 2005 with the REPs from the 2004 database (Haws et al. 2006) for HpCDD, 4-PeCDF and PCB 126. These congeners were chosen based on their suspected deviation from their current WHO-TEFs and their quantitative contribution to the total amount of TEQs in human blood (see Table 1).

### HpCDD

**Fig. 2:**
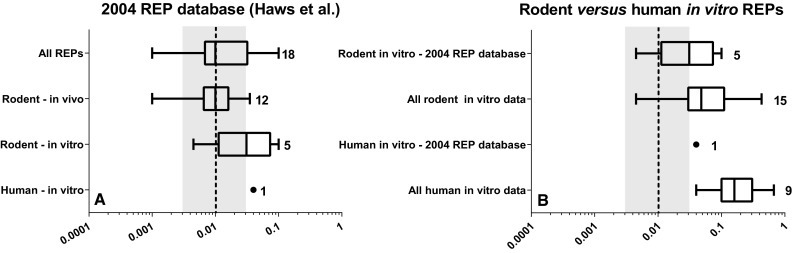
Boxplot comparison of in vivo- and in vitro-derived REPs for HpCDD based on rodent or human data from 2004 REP database (Haws et al. 2006) alone (**a**) and in combination with newly published literature (**b**). Numbers indicate the number of REPs. The *black dashed line* and *gray* area represent the current WHO-TEF value of 0.01 for HpCDD ± half log uncertainty

For HpCDD, the 2004 REP database contains 12 in vivo REPs and 5 in vitro REPs (Haws et al. 2006). The 12 in vivo REPs were all obtained from rat studies and show a median REP of 0.01, which equals the WHO-TEF. The median rodent-based in vitro REP for HpCDD is with 0.03 slightly higher than the median in vivo REP of 0.01 (see Fig. 2a). The 2004 REP database contains only 1 human in vitro REP for HpCDD, which is 0.04 and similar to the median rodent in vitro REP (see Fig. 2a).

Since 2005, two new studies with human and rodent in vitro experiments have been published for this congener providing, respectively, 8 and 10 new REPs (Van Ede et al. 2014b; Larsson et al. 2015). Together with the 2004 REP database, the median rodent and human in vitro REPs are 0.05 and 0.15, respectively, which indicates a possible higher affinity of HpCDD for the human AhR compared with that of rodents. However, there is a broad overlap between the confidence intervals of the rodent and human in vitro REPs (see Fig. 2b). If the information from Fig. 2a, b is combined, two conclusions for HpCDD can be drawn. Rodent in vivo REPs are on average lower than those obtained from in vitro rodent models, which may be explained by lower bioavailability from the GI tract and higher sequestration for this congener compared to TCDD. The median REP of HpCDD in human in vitro models appears a factor three higher than those obtained from comparable rodent in vitro systems, suggesting that HpCDD may be more potent in humans once it is circulating in the body. However, in view of the uncertainties surrounding these HpCDD REPs, such a conclusion should be considered preliminary and must be substantiated further.

### 4-PeCDF

**Fig. 3:**
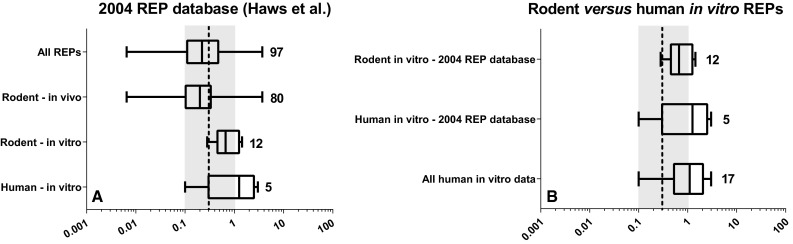
Boxplot comparison of in vivo- and in vitro-derived REPs for 4-PeCDF based on rodent or human data from 2004 REP database (Haws et al. 2006) alone (**a**) and in combination with newly published human REPs (**b**). *Numbers indicate* the number of REPs. The *black dashed line* and *gray* *area* represent the current WHO-TEF value of 0.3 for 4-PeCDF ± half log uncertainty

For 4-PeCDF, the 2004 REP database contains 80 in vivo and 17 in vitro REPs obtained from 20 and 10 studies, respectively (Haws et al. 2006). The in vivo REPs comprise 21 mouse- and 57 rat-based REPs, the latter mainly consisting of in vivo studies from the National Toxicology Program (NTP) using female Sprague–Dawley rats (National Toxicology Program 2006b). The median rodent in vivo REP for 4-PeCDF is 0.2, closely similar to the WHO-TEF of 0.3. In contrast, the median rodent in vitro REP, consisting of 2 mouse and 10 rat REPs, is 0.7, which is higher than the WHO-TEF and the median rodent in vivo REP (see Fig. 3a). The 5 human in vitro REPs from the 2004 REP database were obtained from 2 studies and show a median REP of 1, which is similar to the rodent median in vitro REP and clearly higher than that of the WHO-TEF.

Since 2005, three human in vitro studies published REPs for 4-PeCDF using primary peripheral blood mononuclear cells (PBMCs), hepatocytes, keratinocytes and human hepatoblastoma (HepG2) cells (Budinsky et al. 2010; Van Ede et al. 2014b; Larsson et al. 2015). Combining these new REPs with the existing 2004 database REPs does not change the median REP of 1 for the human in vitro data (see Fig. 3b). As the human in vitro REPs are in line with the rodent-derived in vitro REPs for 4-PeCDF, no obvious toxicodynamic differences for AhR-mediated activities between rodents and humans are present for 4-PeCDF and suggest that for 4-PeCDF there are no species-specific differences. However, a difference between in vivo and in vitro REPs, which is likely due to the higher liver sequestration of 4-PeCDF compared to TCDD, seems apparent.

### Pcb 126

For PCB 126, the WHO-TEF of 0.1 corresponds with the median of 86 in vivo and 29 in vitro REPs obtained from 20 and 19 studies, respectively (Haws et al. 2006). These in vivo REPs comprise of 23 mouse- and 63 rat-based REPs, the latter predominantly consisting of in vivo studies from the NTP using female Sprague–Dawley rats (National Toxicology Program 2006a). REPs for PCB 126 from rat studies are consistently close to 0.1 (Haws et al. 2006). A wider distribution exists for the mouse-based in vivo REPs in this database. The median rodent in vitro REP for PCB 126 is with 0.09 very similar to the median in vivo REP of 0.1.

**Fig. 4:**
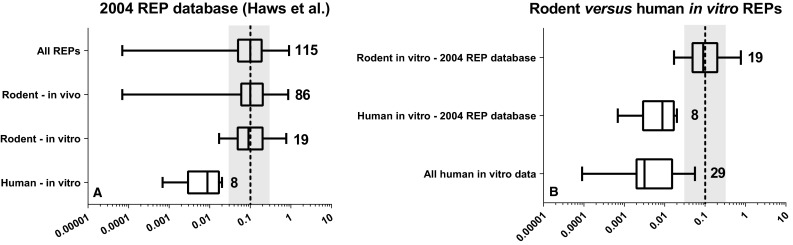
Boxplot comparison of in vivo and in vitro-derived REPs for PCB 126 based on rodent or human data from 2004 REP database (Haws et al. 2006) alone (**a**) and in combination with newly published human REPs (**b**). *Numbers* *indicate* the number of REPs. The *black dashed line* and *gray area* represent the current WHO-TEF value of 0.1 for PCB 126 ± half log uncertainty

Of the 29 in vitro REPs for PCB 126 within this 2004 REP database, 8 are derived from studies using human cells (Drenth et al. 1998; Zeiger et al. 2001; Van Duursen et al. 2003; Pang et al. 1999). Compared with the rodent data, the human in vitro REPs are clearly much lower with a median REP of 0.009 (range 0.0007–0.02) (see Fig. 4). Since 2005, another seven in vitro studies with human primary PBMCs, hepatocytes, keratinocytes or HepG2 cells were conducted that determined the relative potency of PCB 126. These studies show REPs ranging from 0.00009 to 0.06 (Van Duursen et al. 2005; Silkworth et al. 2005; Sutter et al. 2010; Westerink et al. 2008; Carlson et al. 2009; Larsson et al. 2015; Van Ede et al. 2014b). When we combine the newly published data with those from the 2004 REP database, it is evident that in vitro REPs from different human cell systems are consistently one to two orders of magnitude lower than the current WHO-TEF. These data unmistakably show a species-difference in AhR-mediated effects between humans and rodents for PCB 126. As a result, we may consistently be overestimating the toxicity of PCB 126 for humans when using rodent data or the present WHO-TEF.

## Human risk assessment of DLCs in perspective

In order to put the above observations into perspective for human risk assessment, we selected three different studies in which the background blood, plasma or adipose tissue concentrations of DLCs and total TEQs were measured (see Table 1) (Watanabe et al. 2013; Wittsiepe et al. 2007; Rawn et al. 2012). The study of Rawn et al. represents a national baseline estimate of concentrations of DLCs in Canadians (Rawn et al. 2012). The study of Wittsiepe et al. represents the concentrations of DLCs in German mothers living in an industrialized area of Germany (Wittsiepe et al. 2007), while the study of Watanabe et al. represents the concentrations of DLCs in randomly selected deceased patients in Japan (Watanabe et al. 2013).

**Table 1:** Concentrations, TEQ (pg/g lipid) and  % contribution to total TEQ of PCDD/Fs and DLC PCBs in human blood from general populations (Rawn et al. 2012; Wittsiepe et al. 2007; Watanabe et al. 2013)

	WHO-TEF^a^	Human SYS-TEF	Rawn et al. (2012)					Wittsiepe et al. (2007)				
			Mean (pg/g lipid)	Intake-TEQ	% of total Intake-TEQ	HUMAN SYS-TEQ	% of total SYS-TEQ	Mean (pg/g lipid)	Intake-TEQ	% of total Intake-TEQ	HUMAN SYS-TEQ	% of total SYS-TEQ
2378-TCDD	1	1	0.53	0.53	4.0	0.53	3.1	1.3	1.3	6.8	1.3	5.1
12378-PeCDD	1	1	3.7	3.7	28.1	3.7	21.3	4.7	4.7	24.7	4.7	18.5
123478-HxCDD	0.1	0.1	3	0.3	2.3	0.3	1.7	3.8	0.38	2.0	0.38	1.5
123678-HxCDD	0.1	0.1	27	2.7	20.5	2.7	15.6	15	1.5	7.9	1.5	5.9
123789-HxCDD	0.1	0.1	3.7	0.37	2.8	0.37	2.1	3.2	0.32	1.7	0.32	1.3
1234678-HpCDD	0.01	**0.1**	26	0.26	**2.0**	2.6	**15.0**	22	0.22	**1.2**	2.2	**8.6**
OCDD	0.0003	0.0003	180	0.054	0.4	0.054	0.3	220	0.066	0.35	0.066	0.26
2378-TCDF	0.1	0.1	0.61	0.061	0.5	0.061	0.4	0.26	0.026	0.1	0.026	0.1
12378-PeCDF	0.03	0.03	0.35	0.0105	0.08	0.0105	0.06	0.3	0.009	0.0	0.009	0.0
23478-PeCDF	0.3	**1**	5.4	1.62	**12.3**	5.4	**31.1**	12	3.6	**18.9**	12	**47.2**
123478-HxCDF	0.1	0.1	0.42	0.042	0.3	0.042	0.2	4.5	0.45	2.4	0.45	1.8
123678-HxCDF	0.1	0.1	4.1	0.41	3.1	0.41	2.4	4.2	0.42	2.2	0.42	1.7
123789-HxCDF	0.1	0.1	1.3	0.13	0.99	0.13	0.7	0.22	0.022	0.1	0.022	0.1
234678-HxCDF	0.1	0.1	0.35	0.035	0.3	0.035	0.2	1.5	0.15	0.8	0.15	0.6
1234678-HpCDF	0.01	0.01	1.3	0.013	0.10	0.013	0.1	4.7	0.047	0.2	0.047	0.18
1234789-HpCDF	0.01	0.01	0.54	0.0054	0.04	0.0054	0.0	0.31	0.0031	0.0	0.0031	0.0
OCDF	0.0003	0.0003	0.58	0.000174	0.0013	0.00017	0.001	0.65	0.000195	0.0	0.000195	0.00
PCB 77	0.0001	0.0001	10	0.001	0.008	0.001	0.0	7	0.0007	0.0	0.0007	0.003
PCB 81	0.0003	0.0003	10	0.003	0.023	0.003	0.0	1.2	0.00036	0.0	0.00036	0.0
PCB 126	0.1	**0.003**	20	2	**15.2**	0.06	**0.3**	41	4.1	**21.5**	0.123	**0.5**
PCB 169	0.03	0.03	20	0.6	4.6	0.6	3.5	35	1.05	5.5	1.05	4.1
PCB 105	0.00003	0.00003	1100	0.033	0.3	0.033	0.2	1400	0.042	0.22	0.042	0.165
PCB 114	0.00003	0.00003	480	0.0144	0.1	0.0144	0.1	430	0.0129	0.068	0.0129	0.051
PCB 118	0.00003	0.00003	6300	0.189	1.4	0.189	1.1	9200	0.276	1.4	0.276	1.08
PCB 123	0.00003	0.00003	60	0.0018	0.01	0.0018	0.01	130	0.0039	0.0	0.0039	0.0
PCB 156	0.00003	0.00003	400	0.012	0.1	0.012	0.1	7200	0.216	1.1	0.216	0.8
PCB 157	0.00003	0.00003	940	0.0282	0.2	0.0282	0.2	1100	0.033	0.2	0.033	0.1
PCB 167	0.00003	0.00003	990	0.0297	0.2	0.0297	0.2	2100	0.063	0.3	0.063	0.2
PCB 189	0.00003	0.00003	370	0.0111	0.1	0.0111	0.1	1100	0.033	0.17	0.033	0.13
Total PCDD-TEQ				7.9	60.1	10.3	59.1		8.5	44.6	10.5	41.1
Total PCDF-TEQ				2.3	17.7	6.1	35.2		4.7	24.8	13.1	51.6
Total non-*ortho*-PCBs-TEQ			2.6	19.8	0.7	3.8		5.2	27.0	1.2	4.6	
Total mono-*ortho*-PCBs-TEQ			0.32	2.4	0.3	1.8		0.7	3.6	0.7	2.7	
Total TEQs				13.2	100	17.3	100		19.0	100.0	25.4	100

	Watanabe et al. (2013)				
	Mean (pg/g lipid)	Intake-TEQ	% of total Intake-TEQ	HUMAN SYS-TEQ	% of total SYS-TEQ
2378-TCDD	3.3	3.3	4.7	3.3	4.8
12378-PeCDD	15	15	21.4	15	21.6
123478-HxCDD	5.6	0.56	0.8	0.56	0.8
123678-HxCDD	71	7.1	10.1	7.1	10.2
123789-HxCDD	7.7	0.77	1.1	0.77	1.1
1234678-HpCDD	16	0.16	**0.2**	1.6	**2.3**
OCDD	320	0.096	0.1	0.096	0.1
2378-TCDF	1.4	0.14	0.2	0.14	0.2
12378-PeCDF	1	0.03	0.0	0.03	0.0
23478-PeCDF	30	9	**12.8**	30	**43.3**
123478-HxCDF	6.7	0.67	1.0	0.67	1.0
123678-HxCDF	7.8	0.78	1.1	0.78	1.1
123789-HxCDF	0.2	0.02	0.0	0.02	0.0
234678-HxCDF	1.8	0.18	0.3	0.18	0.3
1234678-HpCDF	2.6	0.026	0.0	0.026	0.0
1234789-HpCDF	0.5	0.005	0.0	0.005	0.0
OCDF	1	0.0003	0.0	0.0003	0.0
PCB 77	6.3	0.00063	0.001	0.00063	0.0
PCB 81	7.4	0.00222	0.003	0.00222	0.0
PCB 126	240	24	**34.2**	0.72	**1.0**
PCB 169	220	6.6	9.4	6.6	9.5
PCB 105	5000	0.15	0.2	0.15	0.2
PCB 114	1900	0.057	0.1	0.057	0.1
PCB 118	24,000	0.72	1.0	0.72	1.0
PCB 123	480	0.0144	0.0	0.0144	0.0
PCB 156	15,000	0.45	0.6	0.45	0.6
PCB 157	3500	0.105	0.1	0.105	0.2
PCB 167	4900	0.147	0.2	0.147	0.2
PCB 189	2700	0.081	0.1	0.081	0.1
Total PCDD-TEQ		27.0	38.5	28.4	41.0
Total PCDF-TEQ		10.9	15.5	31.9	45.9
Total non-*ortho*-PCBs-TEQ	30.6	43.6	7.3	10.6	
Total mono-*ortho*-PCBs-TEQ	1.7	2.5	1.7	2.5	
Total TEQs		70.2	100.0	69.3	100

^a^Current WHO-TEF (Van den Berg et al. 2006)

### Dioxins

It is of interest to note that after PeCDD, 1,2,3,6,7,8-hexachlorodibenzo-*p*-dioxin (6-HxCDD) is the second most important contributor to the total amount of TEQs in the general population based on the current WHO-TEFs (see Table 1), accounting for approximately 20, 8 and 10 % of the TEQ in the Canadian, German and Japanese background levels (Wittsiepe et al. 2007; Watanabe et al. 2013; Rawn et al. 2012). In comparison, the quantitative contribution of HpCDD is approximately 0.2–2 % of the total amount of TEQs using the current WHO-TEFs. However, if we look at the results of our evaluation, the median rodent and human in vitro REP for HpCDD is 0.1, and our data indicate that such a value would be more appropriate for a systemic REP. Such a higher value for HpCDD significantly increases its contribution to the total TEQ measured in blood or adipose tissue in the different populations. As a result, HpCDD would become one of the major contributors to the total amount of TEQs in the blood (for comparisons, see Table 1) (Wittsiepe et al. 2007; Watanabe et al. 2013; Rawn et al. 2012). It should also be noted that for the other dioxins with WHO-TEF values, the 2004 REP database does not show a clear deviation between in vitro and in vivo or human and rodent data. For the human risk assessment of dioxins based on blood levels, it would mean that only a separate systemic TEF for HpCDD is recommended (for comparison, see Table 2) (Haws et al. 2006).

**Table 2:** Summary evaluation of WHO-TEF congeners for in vitro versus in vivo and human vs. rodent differences, and proposed human systemic TEF (SYS-TEF) values, if different from WHO-TEF

Congener	WHO-TEF^a^	Evidence for Differences				
		In vitro versus in vivo	Human versus rodent	Proposed human SYS-TEF	REF^b^	Comments
Chlorinated dibenzo-*p*-dioxins						
2378-TCDD	1					
12378-PeCDD	1					
123478-HxCDD	0.1					
123678-HxCDD	0.1					
123789-HxCDD	0.1					
1234678-HpCDD	0.01	Yes		0.1	^5, 6, 12^	See Fig. 2
OCDD	0.0003					
Chlorinated dibenzofurans						
2378-TCDF	0.1	Yes			^3, 5^	No proposed SYS-TEF as mean REP is similar to WHO-TEF (see Fig. 1)
12378-PeCDF	0.03	Yes		0.1	^3, 5^	See Fig. 1
23478-PeCDF	0.3	Yes		1	^1, 3, 5, 6, 10, 11, 12^	See Fig. 3
123478-HxCDF	0.1	Yes	Possibly	1	^5, 6, 12^	
123678-HxCDF	0.1		Possibly		^8^	Scarce data
123789-HxCDF	0.1					
234678-HxCDF	0.1					
1234678-HpCDF	0.01					
1234789-HpCDF	0.01	Yes		0.3	^5, 6, 12^	
OCDF	0.0003	Yes		0.001	^3, 5^	See Fig. 1
Non-*ortho*-substituted PCBs						
PCB 77	0.0001		Possibly		^4, 6, 12, 14^	No CYP1A1 induction at all or high enough to calculate REP in human cells
PCB 81	0.0003		Possibly		^14^	No CYP1A1 induction at all or high enough to calculate REP in human cells
PCB 126	0.1		Yes	0.03	^2, 5, 6, 7, 8, 9, 12, 13^	See Fig. 4
PCB 169	0.03		Possibly		^6, 12, 14^	No CYP1A1 induction at all or high enough to calculate REP in human cells
Mono-*ortho*-substituted PCBs						
PCB 105	0.00003		Possibly		^6, 12, 14^	No CYP1A1 induction at all or high enough to calculate REP in human cells
PCB 114	0.00003		Possibly		^6, 12, 14^	No CYP1A1 induction at all or high enough to calculate REP in human cells
PCB 118	0.00003		Possibly		^4, 6, 12, 14^	No CYP1A1 induction at all or high enough to calculate REP in human cells
PCB 156	0.00003		Possibly		^6, 12, 14^	No CYP1A1 induction at all or high enough to calculate REP in human cells
PCB 157	0.00003		Possibly		^6, 12, 14^	No CYP1A1 induction at all or high enough to calculate REP in human cells
PCB 167	0.00003		Possibly		^6, 12, 14^	No CYP1A1 induction at all or high enough to calculate REP in human cells
PCB 189	0.00003		Possibly		^6, 12, 14^	No CYP1A1 induction at all or high enough to calculate REP in human cells

^a^ Current WHO-TEF (Van den Berg et al. 2006)

^b^ ^1^ Budinsky et al. (2010), ^2^ Carlson et al. (2009), ^3^ DeVito et al. (1997), ^4^ Endo et al. (2003), ^5^ Haws et al. (2006), ^6^ Larsson et al. (2015), ^7^ Silkworth et al. (2005), ^8^ Sutter et al. (2010), ^9^ Van Duursen et al. (2005), ^10^ Van Ede et al. (2013), ^11^ Van Ede et al. (2014a), ^12^ Van Ede et al. (2014b), ^13^ Westerink et al. (2008), ^14^ Zeiger et al. (2001)

### Furans

Of the furans, in particular 4-PeCDF is a substantial contributor to the total TEQs in the human population. Our review shows that the median systemic REP as well as rodent and human in vitro REPs is higher than the rodent median in vivo REP and the present WHO-TEF. However, the deviation found is mostly within the half log uncertainty around the WHO-TEF of 0.3 (see Figs. 1, 3). Nevertheless, it should be noted that the 75th percentile of the systemic REP distribution and the 25th to 75th percentile REP distribution for rodent and human in vitro REPs are above the current WHO-TEF for 4-PeCDF.^1^ If a systemic TEF of 1 for 4-PeCDF is applied for the systemic blood and adipose concentrations in the different populations, this would result in an even stronger contribution of this congener to the total amount of TEQs (for comparison, see Table 1) (Wittsiepe et al. 2007; Watanabe et al. 2013; Rawn et al. 2012).

For TCDF, 1-PeCDF, 4-HxCDF, 6-HxCDF, 9-HpCDF and OCDF, differences between in vitro and in vivo or between human and rodent REPs were also observed (see Table 2). However, the quantitative contributions of these congeners, except for 4-HxCDF and 6-HxCDF, are very low in blood and will have little impact on total TEQs in the general population (see Table 1) (Wittsiepe et al. 2007; Watanabe et al. 2013; Rawn et al. 2012). For 4-HxCDF higher human and rodent in vitro REPs with a median of, respectively, 1.5 and 0.3 were seen compared to the median in vivo REP of 0.05 or WHO-TEF of 0.1 (Larsson et al. 2015; Haws et al. 2006, Van Ede et al. 2014b). In addition, a higher human in vitro REP of 1 for 6-HxCDF was determined in human keratinocytes compared to the in vitro and in vivo rodent REPs as well as the WHO-TEF of 0.1 (Sutter et al. 2010). Applying a higher systemic or human-specific TEF value for these two congeners has significant implications, as the contribution to the total TEQs based on their current WHO-TEFs is already above 1 % (see Table 1) (Wittsiepe et al. 2007; Watanabe et al. 2013; Rawn et al. 2012).

### PCBs

For PCB 126, the combined human REP data from the literature, shown in Fig. 4, gives a median human REP of 0.003. This is almost a 100 times lower than the WHO-TEF of 0.1, and far outside its suggested ± half log uncertainty range. PCB 126 is one of the major contributors to the total amount of TEQs in human blood when using the present WHO-TEFs. If the TEF for PCB 126 would be adjusted for humans from 0.1 to 0.003, the contribution of PCB 126 to the total TEQ goes from 15–35 to 1–2 % and becomes negligible (see Table 1) (Wittsiepe et al. 2007; Watanabe et al. 2013; Rawn et al. 2012).

In contrast to dioxins and furans, multiple experimental studies indicate that humans may be less responsive to PCB congeners that are (relatively) potent AhR agonists in rodents. As can be seen from Fig. 4b, this is clearly the case for the non-*ortho*-substituted PCB 126, but it also applies for others such as PCB 169 and mono-*ortho*-PCBs. Especially the latter group of PCB congeners has been shown a lack of capability to induce a significant dioxin-like response in human primary cells or cell lines (see Table 2) (Zeiger et al. 2001; Endo et al. 2003; Larsson et al. 2015; Van Ede et al. 2014b). The clear difference in response between these PCBs versus PCDDs and PCDFs in human systems compared with those of rodents most likely originates from species-specific binding properties to the AhR, which specifically makes humans less sensitive to PCBs (Petkov et al. 2010).

### Effect on total TEQ

Our review of the more recent scientific data indicates that it may be appropriate to use human-specific or systemic TEFs instead of intake WHO-TEFs for human risk assessment for 6-HpCDD, 4-PeCDF and PCB 126, when based on systemic concentrations in blood or tissues. In Table 2, some suggestions for TEF changes are given for these congeners. If such a change would be adapted, it would change the total amount of TEQs in human blood. Such a change would result in an increase in the relative contribution to the total TEQs by PCDDs and PCDFs of 5–23 and 62–66 %, respectively, and a 75–77 % decrease in contribution of total TEQs by non-*ortho*-PCBs (Table 1) (Wittsiepe et al. 2007; Rawn et al. 2012; Watanabe et al. 2013). Overall, the effect of such TEF changes for these three congeners on total TEQ would roughly balance each other out in the general population. However, results may be different for those situations in which a specific group of congeners dominates, e.g, a food contamination incident or under conditions of unusual occupational exposures. Furthermore, also the effects of for instance 4-HxCDF or 6-HxCDF are not taken into account.

The above systemic TEQ estimates that were calculated using the adjusted systemic TEF values can be further evaluated in a risk assessment context. Previously, Biomonitoring Equivalents (BEs), which are estimates of steady-state biomarker concentrations consistent with chronic exposure at a reference dose (RfD) or tolerable daily intake (TDI), have been estimated for TCDD (Aylward et al. 2008, 2013). The physiologically based pharmacokinetic (PBPK) model for TCDD selected by USEPA in their 2012 RfD evaluation was used to estimate steady-state serum TCDD concentrations consistent with chronic exposure at the USEPA reference dose (RfD) or the WHO JECFA TDI. The USEPA chronic RfD for TCDD is 0.7 pg TEQ/kg-d. The corresponding BE_RfD_ values for serum concentrations from chronic exposure at this rate are approximately 15 pg TEQ/g lipid for adolescents, rising to approximately 21 pg TEQ/g lipid for adults. Calculated systemic TEQ concentrations can be compared directly to the BE values to evaluate whether the chronic population exposure is well below, near or above the exposure guidance values. The calculated systemic TEQ values, using the adjusted systemic TEF values, for the average population presented in Table 1 are near (Rawn et al. 2012) or above the BE_RfD_ (Wittsiepe et al. 2007; Watanabe et al. 2013). The JECFA TDI is approximately 2.3 pg TEQ/kg-d, leading to a BE_TDI_ of approximately 40–70 pg TEQ/g lipid, depending on assumptions regarding body composition. All three populations mean values fall within or below this range.

## Conclusions and recommendations

Selection of TEF values for dioxin-like compounds that are most appropriate and accurate for their intended applications is important for human risk assessment. For the WHO-TEF methodology, rodent studies based on an administered dose have been considered most suitable for human risk assessment. However, human exposure to DLCs is often reported based on blood or tissue levels. In this review, we have addressed the question to which extent the present intake-based WHO-TEFs are also useful for studies reporting systemic concentration of DLCs, e.g., in blood. It can be proposed that the use of systemic REPs with results from human in vitro studies may provide a more accurate human risk assessment, because it will bridge the gap in toxicokinetics and toxicodynamics between species. Our review shows that in vitro REPs of some congeners may well represent systemic REPs. Thus, in vitro REPs may provide a reasonable basis for assessing systemic as well as human-specific REPs. Based on the existing data that have been used in this review, a number of specific conclusions can be drawn:

- Both rodent and human in vitro REPs for 6-HpCDD are up to one order of magnitude higher than their current WHO-TEFs, with human in vitro data tending to have a higher relative potency than the rodent in vitro data (Fig. 2).
- Rodent and human in vitro REPs for 4-PeCDF span a similar range and indicate that this congener is more potent on a systemic basis than on an intake basis, perhaps by as much as one order of magnitude (Fig. 3).
- Combining REPs from the 2004 REP database with newly published data demonstrates that the human-based in vitro REP for PCB 126 is at least one, but possibly up to two orders of magnitude lower than expected based on the current WHO-TEF (Fig. 4).

For those congeners that show a distinct deviation between either intake and systemic REPs or between rodent- and human-based in vitro REPs, we propose that in vitro REP data are weighted more heavily in establishing systemic or human-specific TEF values. Especially REPs derived from human-based in vitro models should be considered more valuable contributors to improve human health risk assessment for dioxin-like compounds.

## Abbreviations

AhR: Aryl hydrocarbon receptor
BEs: Biomonitoring Equivalents
DLC: Dioxin-like compound
GI: Gastrointestinal
HepG2: Human hepatoblastoma cells
HpCDD: 1,2,3,4,6,7,8-Heptachlorodibenzo-*p*-dioxin
6-HxCDD: 1,2,3,6,7,8-Hexachlorodibenzo-*p*-dioxin
9-HpCDF: 1,2,3,4,7,8,9-Heptachlorodibenzofuran
4-HxCDF: 1,2,3,4,7,8-Hexachlorodibenzofuran
6-HxCDF: 1,2,3,6,7,8-Hexachlorodibenzofuran
NTP: National toxicology program
OCDF: 1,2,3,4,6,7,8,9-Octachlorodibenzofuran
PBMCs: Peripheral blood mononuclear cells
PCB: Polychlorinated biphenyl
PCB 126: 3,3′,4,4′,5-Pentachlorobiphenyl
PCDD: Polychlorinated dibenzo-*p*-dioxin
PeCDD: 1,2,3,7,8-Pentachlorodibenzo-*p*-dioxin
PCDF: Polychlorinated dibenzofuran
1-PeCDF: 1,2,3,7,8-Pentachlorodibenzofuran
4-PeCDF: 2,3,4,7,8-Pentachlorodibenzofuran
REP: Relative effect potencies
RfD: Reference dose
TCDD: 2,3,7,8-Tetrachlorodibenzo-*p*-dioxin
TCDF: 2,3,7,8-Tetrachlorodibenzofuran
TEF: Toxic equivalency factor
TEQ: Toxic equivalency
TDI: Tolerable daily intake
WHO: World Health Organization

## Footnotes

1 The 75th percentile was used during the 2006 expert meeting as a cutoff point for reevaluation.
